# Development of a decision aid to support colorectal cancer screening: perspectives of Asians in an endemic urban community—a qualitative research study

**DOI:** 10.1186/s12911-021-01404-1

**Published:** 2021-03-06

**Authors:** Sok Wei Julia Yuen, Tsang Yew Tay, Ning Gao, Nian Qin Tho, Ngiap Chuan Tan

**Affiliations:** 1 SingHealth Polyclinics, 167, Jalan Bukit Merah, Connection One, Tower 5, #15-10, Singapore, 150167 Singapore; 2 Ministry of Health Holdings, Singapore, Singapore; 3 SingHealth-Duke NUS Family Medicine Academic Clinical Programme, Singapore, Singapore

**Keywords:** Colorectal cancer, Screening, Decision aid, Design, Content, Utility

## Abstract

**Background:**

Colorectal cancer (CRC) is a common malignancy worldwide. Despite being the most common cancer in Singapore, CRC screening rate remains low due to knowledge deficits, social reasons such as inconvenience and a lack of reminder or recommendation. A decision aid (DA) may facilitate an individual’s decision-making to undertake CRC screening by addressing misconceptions and barriers. We postulate that a more person-centred and culturally adapted DA will better serve the local population. The views of the target users are thus needed to develop such a DA. A CRC screening DA prototype has been adapted from an American DA to cater to the Asian users. This study aimed to explore user perspectives on an adapted CRC screening DA-prototype in terms of the design, content and perceived utility.

**Methods:**

The study used in-depth interviews (IDIs) and focus group discussions (FGDs) to gather qualitative data from English-literate multi-ethnic Asian adults aged 50 years old and above. They had yet to screen for CRC before they were recruited from a public primary care clinic in Singapore. The interviews were audio-recorded, transcribed and analysed to identify emergent themes via thematic analysis.

**Results:**

This study included 27 participants involved in 5 IDI and 5 FGDs. Participants found the DA easily comprehensible and of appropriate length. They appreciated information about the options and proposed having multi-lingual DAs. The design, in terms of the layout, size and font, was well-accepted but there were suggestions to digitalize the DA. Participants felt that the visuals were useful but there were concerns about modesty due to the realism of the illustration. They would use the DA for information-sharing with their family and for discussion with their doctor for decision making. They preferred the doctor’s recommendation for CRC screening and initiating the use of the DA.

**Conclusions:**

Participants generally had favourable perceptions of the DA-prototype. A revised DA will be developed based on their feedback. Further input from doctors on the revised DA will be obtained before assessing its effectiveness to increase CRC screening rate in a randomized controlled trial.

## Background

Colorectal cancer (CRC) is prevalent worldwide, with an age-standardized rate as high as 51.2 per 100,000 in Hungary [1]. Singapore ranks twelfth globally, with CRC affecting 38.6 males and 27.0 females per 100,000 on this urbanised island-state. The Singapore Cancer Registry Annual Report 2015 [2] reported a total of 9807 new CRC cases from 2011 to 2015.

The adenoma-carcinoma sequence takes approximately 10 years. This long period of progression makes it ideal for CRC screening [3]. Prospective randomised control trials (RCTs) have shown a reduction in CRC mortality by 15– 33% with CRC screening using faecal occult blood test (FOBT) [4] 56. This reduction in mortality is due to early detection, removal of pre-malignant adenomas by colonoscopy and early treatment [7].

Like other international CRC screening guidelines [8], the Singapore guidelines recommend that CRC screening begins at age 50 years [9]. The recommended population-based screening test for an average risk individual is the FOBT annually or the colonoscopy once every decade. Other screening tests include flexible sigmoidoscopy and computed tomographic (CT) colonography [9].

Nevertheless, CRC screening rate in Singapore remains low. The 2010 Singapore National Health Survey revealed that only 19.7% of Singapore residents aged 50–69 years old had either FOBT within the past 1 year or colonoscopy/sigmoidoscopy within the past 10 years [10]. Another local study in 2013 showed that the CRC screening rate among community dwelling participants aged 50 years and older was 26.7%. The most common modality was FOBT, followed by colonoscopy [11]. The major barriers to local FOBT screening include paucity of symptoms, absence of family history of CRC and social reasons such as inconvenience; lack of time, reminders and recommendations. The barriers to colonoscopy were similar, with the additional concern about cost [12]. A RCT conducted by Chua et al. [13] reported that participants were more willing to do the FOBT with education and family physician recommendation, highlighting the pivotal role of the physician in promoting CRC screening.

The asymptomatic individual of average risk may face difficulties deciding on a particular screening modality due to the various available options. This creates a challenge for the family physician as it takes time and effort to convey information about CRC, the importance of screening, the options available and the risks and benefits of each option. Ideally, the physician has to weigh the individual’s values regarding the potential benefits and harms associated with CRC screening, deliberate on the individual’s preferences before arriving at a joint decision [14].

Decision aids (DAs) are evidence-based support tools that improve an individual’s knowledge about available options, consider their personal values and preferences and promote a more active role in Shared Decision Making [15]. RCTs done in the United States (US) showed that DAs used for CRC screening increased patient’s knowledge, intent and decision for screening [16]^17^. According to the Ottawa Decision Support Framework (ODSF), DAs assist an individual to make quality decisions by addressing their decisional needs [18]. It can be deployed before, during and after the physician’s consultation.

Conventionally, the development of DAs can consume considerable manpower, time and costs [19]. Existing CRC screening DAs have been created in Australia and the US. These can be found on the Ottawa Hospital Research Institute website [20]. Adapting DAs rather than developing completely new ones can save time and costs. However, during the process of preparing it for use in a different setting from where it was originally created, it is important that the DA be contextualized to the specific needs, cultural values and preferences of the target population [21].

Chenel et al. [22] has identified and described the four main phases in the process of cultural adaptation of DAs, namely exploration, adaptation, lab testing and field testing. This study focused on the exploration and adaptation of the DA to suit the information needs, values and preferences of Asian adults towards colorectal cancer screening. In the exploration phase, it is important to critically appraise the original DA to ensure that it is of high quality, as well as the new cultural context to better understand what needs to be adapted. In the adaptation phase, the focus is to ensure that the wording and the content of the DA fit the local context. This is achieved by gathering end users’ feedback on the draft DA.

In this study, the investigators have adapted and modified an American CRC screening DA to create a novel DA-prototype for the local population. Singapore’s local population is largely Asian, consisting of 76% Chinese, 15% Malays and 7.5% Indians [23]. Its acceptability by the target users is crucial to its successful implementation in clinical practice. Thus, gathering their feedback on the prototype is essential in the development of the final DA.

## Methods

### Aim

This study aimed to explore the perspectives of multi-ethnic English literate Asian adults on the design, content and perceived utility of the CRC screening DA-prototype. The investigators postulated that their views and feedback would contextualize the DA-prototype, so that the eventual DA would effectively cater to their needs, address their concerns and enable them to make quality decisions on CRC screening.

### Study design and conceptual framework

Qualitative research method was used to explore the perspectives of Asian adults on the CRC screening DA-prototype via in-depth interviews (IDIs) and focus group discussions (FGDs).

The ODSF underpins the conceptual framework of the study [18]. It is a support framework to address decisional uncertainty by providing decision support in order for the user to make a quality and informed decision.

Low CRC screening rates in Singapore is a result of decisional uncertainty, contributed by a plethora of factors as mentioned above. In accordance with the ODSF framework, decision support can be provided in a few avenues, such as a decision tool, which can provide facts, clarify needs and decisions and assist in communication and deliberation. The DA, adapted to cultural needs, aims to fulfil this role as a decision support tool towards promoting SDM between the patient and clinician, which should in turn improve the CRC screening rate.

### Development of the novel CRC screening DA-prototype

The investigators started by exploring the available CRC screening DAs available on the Ottawa Hospital Research Institute website and from literature search. Of these, the investigators have chosen to adapt the one developed by Healthwise from the US [24]. It satisfies the majority of the International Patient Decision Aid Standards (IPDAS) [25], an approved set of criteria to guide the development of and assess the quality of DAs. Using the Simply Put guide [26], the investigators endeavoured to ensure that the content, text appearance, visuals, layout and design were easily understood.

The prototype is a 23-paged, coloured A5 sized booklet printed in English, the lingua franca of Singapore. The DA-prototype is divided into 5 sections: Introduction to CRC screening, What are your options, Comparing your options, What matters most to you and What is your decision.

Known local barriers to CRC screening were addressed in the introduction. Visuals of available screening options were incorporated under “What are your options”. Approval was sought from Singapore Cancer Society to use illustrations from their website [27]. The DA-prototype included a bipolar scale in the section “What matters most to you?”, adapted from Healthwise. The user indicates on the scale how important two contrasting statements are to them, with the extremes favouring FOBT or colonoscopy/others. The midpoint indicates that both statements are equally important. For example, “I prefer to do the test at home in private” favours the FOBT versus “I do not mind going to the hospital to have the test done” favours the colonoscopy/others.

### Topic guide

A topic guide was created to interview the participants in this study, focusing on the content, design and perceived utility of the DA (see Additional file 1: Appendix 1: topic guide).

### Study site

The study site was at Tampines Polyclinic, a public primary care clinic located in Eastern Singapore. The polyclinic provides comprehensive primary care services to approximately 261,230 multi-ethnic Asian residents, comprising 67.1% Chinese, 21.4% Malays and 8.3% Indians [28]. The interviews were conducted in English from February 2019 to February 2020.

### Study population

Purposive sampling was carried out to enrol average risk multi-ethnic Asians, aged 50 years and older, who had not done up-to-date CRC screening. They should be able to understand, retain, weigh the information provided in the DA-prototype and communicate their views in English during the interviews.

Participants who had self-reported histories of colonic polyps, inflammatory bowel disease (IBD) or CRC, or had a first degree relative diagnosed with CRC, were excluded. Those who had undifferentiated bowel signs and symptoms were also excluded. These high risk individuals are recommended for CRC screening before 50 years old, undergo more frequent screening or get specific tests.

### Recruitment

The investigators approached potential participants at the study site. Participants were recruited if they satisfied the eligibility criteria and provided written consent. They were given the Participant Information Sheet and their queries on the study intent and protocol were addressed. They were also given the DA-prototype and encouraged to review it in detail prior to the interviews. Participants were notified of the date and time of their scheduled interviews.

### Interviews

The interviews were moderated by investigator YSWJ. YSWJ is a Family Physician with a Masters in Family Medicine and has been trained in qualitative research. Participants were not known to YSWJ prior to the interviews. During the pre-interview briefing, confidentiality was emphasized. IDIs were conducted to test the flow of the questions in the topic guide. This allowed the investigators to assess if the participants were able to freely share their perspectives without fear of being judged. FGDs were subsequently conducted on participants with diverse profiles to gather a wide spectrum of perspectives. They were provided opportunities to exchange viewpoints and discuss areas of disagreements. The duration of each IDI and FGD was about 30 and 60 min respectively.

The interviews were conducted in a quiet conference room at the polyclinic to ensure privacy. Participants were anonymized and addressed by their study identification. They were reimbursed with vouchers worth thirty Singapore dollars (SGD) [approximately twenty two United States dollars (USD)] for their time and travel expenses. The interviews were audio-recorded, transcribed verbatim by either a study team member or a professional transcriber with reference to field notes. The transcribed texts were audited by YSWJ with reference to the audio recordings and errors were corrected.

### Data coding

After reading the transcripts from the initial two IDIs, two investigators YSWJ and TNQ independently coded the data. While the investigators did not formally assess the inter-rater reliability, there was a high level of agreement between the coders when they convened. There were only a few differences, which were deliberated between the coders to reach a consensus. The generation of the initial set of codes helped to guide the coding of subsequently gathered data. As more interviews were completed, new codes that surfaced were discussed among investigators and added to the codebook.

### Data analysis

Thematic analysis was used. As described by Braun and Clarke [29], it emphasizes six key steps; familiarizing with data, generating codes, searching for themes, reviewing these themes, defining them and finally producing the report. The analysis was conducted in an iterative approach, taking into account participant perspectives as well as reflections and inductions from the investigator. In addition, deliberation on the themes was carried out with other co-investigators. The interviews were terminated when no new theme was identified. Thematic saturation was reached after five IDIs and five FGDs.

Participant details and consent forms, recordings, field notes, transcripts and coding were organized in secure archives to ensure a clear audit trail.

### Ethics approval and funding

The study received ethics approval from the SingHealth centralized institutional review board (CIRB reference: 2018/3232). It was sponsored by the SingHealth Seed Fund (SHP-SEED50-2019).

## Results

27 participants, comprising 10 men and 17 women, aged between 50 and 77 years from all major ethnicities in Singapore, were interviewed (Table 1).

**Table 1:** Demographic characteristics of the study population

Demographic characteristics	Participants (n = 27)
Gender	
Male	10
Female	17
Age (years)	
50–59	11
60–69	12
70 and above	4
Ethnicity	
Chinese	17
Malay	7
Indian	2
Others: Eurasian	1
Education	
Primary	1
Secondary	9
Post-secondary or diploma	13
Post tertiary or University	4
Employment status	
Homemaker	5
Employed	14
Unemployed	2
Retired	6

The investigators presented the results according to the following domains; perspectives on the content, design of the DA-prototype and its perceived utility.

### Content

#### Relevance and volume of the content

Despite the varying educational qualifications, the majority of participants found the content easily comprehensible and of appropriate length. Only a minority of the participants in the FGDs felt that the DA was too lengthy. They preferred it to be shortened to four to five pages from the current 23 pages. Most of them agreed that the information was relevant and useful in assisting them to make a decision. It also addressed most of their concerns about CRC screening. Some sought clarification on the content; an example was what constituted a significant family history as a risk factor for CRC.

“Easy to understand… More information is good. People like us are not medical or very educated, we don’t know anything.” P10, employee with diploma education

“Contents are good. It tells you everything you need to know. It’s self-explanatory… This is very good. It shows you your options, what you can do about it.” P29, diploma education

#### Understanding screening options and risks

During the FGDs, it was evident that while majority had heard of CRC screening, they were unaware of all the options available. After going through the DA, participants recognised and understood the locally available screening options and their associated risks. These were knowledge that was previously unknown to them. They became aware of the accessibility of FOBT, and were surprised that they could collect free faecal immunochemical test (FIT) kits from various community service points such as pharmacies. The diagram from Singapore Cancer Society further aided their understanding on how to properly collect a stool sample without contamination. Though intimidating to a minority, most participants wanted to be told of the risks of the various screening options.

“Until I look at this booklet then I’m fully aware there are a few ways (screening options).” P4

“We must know all the options so that we can choose the suitable one for us… I prefer to know the risks of options but I’m also scared.” P9, homemaker, on being asked if she wanted to know all the options and their risks and benefits

They sought practical information on how to obtain an appointment for colonoscopy, an invasive procedure performed by specialists in the hospital.

“If I want to go for colonoscopy, what is my first step? This part, I’m not sure. That means, I walk in to the hospital and say I want to do it?” P32, tertiary educated, freely mentioned due to an interest in doing the colonoscopy

The term “doctor’s office”, while common in North America appeared alien to local participants, even those with tertiary education. They preferred nomenclature like “clinic” instead. They were also unfamiliar with medical jargon, such as IBD, which is listed as a risk factor.

“It’s stated there “test done in doctor’s office”. Office? Is it? How about “clinic” kind of thing?” P26, tertiary educated

#### Understanding the section on “what matters most to you?”

Participants from a few FGDs had difficulty understanding and required guidance to navigate this section. There were suggestions to simplify this section.

“This part must be done with someone who can understand. Medical person or something. It can’t be done alone if you don’t understand.” P23, diploma education

#### Multilingual DAs

Participants suggested having the DA in various languages such as Mandarin, Malay and Tamil in order to cater to the multi-ethnic population in Singapore. This sentiment was common throughout the IDIs and FGDs.

“It’s only one language, English, what happen to the other languages? And these people sometimes don’t understand English much. If you put in 2 or 3 languages, might be easier for them.” P2, secondary educated

### Design

#### Presentation and readability

Participants accepted the size, layout, font and font size of the DA. They suggested having pictures on the cover page to make the DA more attractive and increasing the size of some visuals so that the accompanying text was more legible.

“For the size, I think it’s easy for them to tuck into a plastic bag…” P2

“I think the layout is good… I can read it without my glasses.” P4, retired

#### Digitalizing

There were mixed opinions about digitalizing the DA. Some felt that it would be useful, as most people owned smartphones. Others were concerned that the target age group may not be tech-savvy and preferred the printed version.

“I would say it’s useful because nowadays everybody owns a hand-phone, they can just share around. Of course, the booklet, it does serve its own purpose, especially those who do not download the app, they won’t get to see it.” P26, tertiary educated

#### Visuals

The visuals were useful as they enhanced participants’ understanding of the screening procedures. However, in one of the FGDs, a female participant raised her concerns about modesty with regards to the graphic of the colonoscopy in the draft DA. Although the other participants did not initially share the same concern, they concurred on the sensitivity of the graphic in the multi-religious and cultural context of the local population. They suggested hand drawn or cartoon-like visuals instead.

“Maybe hand drawn, so at least it’s not so graphic and so very real? … we have that element of realness there, but can you make it like a little friendlier? And also, like, modesty wise” P18, Muslim participant

“For me, I’m okay, because I have an open mind, but as for some other religions and other communities, I’ll respect their view.” P30, in response to P18’s concerns about modesty

### Utility

#### Decision making with doctor

Most participants wanted to be told of all the available options. They expressed their preference to be involved in the discussion with their doctor before making a decision regarding screening. A minority preferred to leave the decision making to the doctor, trusting that the doctor would make the best decision for them. Others felt that it depended on the decision to be made, preferring to make their own decision with regards to screening and having shared decision making with their doctor if they were having symptoms and needed medical advice.

“We can discuss and see which is the best. More comfortable before going for it.” P15 expressing her preference for discussing with the doctor before making a decision

They understood the time constraints of a doctor’s consultation in a busy public primary care clinic. It would be challenging to go through the amount of information in the DA within one consultation and also to retain all the relayed information. They were willing to read the DA in detail at home and seek clarification with the doctor at the next visit. Most still preferred a face-to-face discussion with the doctor for further explanation.

“Any queries and concerns, we can go back and ask him (doctor). I don’t think they have time to one-by-one explain, because it’s not fair to the patient (waiting) behind (referring to queue).” P31

They highlighted that the doctor would be the key healthcare professional to recommend CRC screening. They valued the doctor’s opinion and perceived it to be more impactful if the doctor were to give them the CRC screening DA over other healthcare professionals such as nurses or ancillary stuff.

“If the doctor gives, it’s better. It’s more effective than being given by the nurse.” P30

“Because we’ve just been checked by the doctor, of course we trust the doctor more. He had just, you know, looked at our medical history, then he gives us this, of course we will trust that.” P18, Diploma education, agreeing with P30

#### Personal decision-making for CRC screening

Participants self-reported increased intent for screening after going through the DA. Some reasons cited include the new found knowledge about how prevalent CRC is in Singapore and the asymptomatic phase of disease. A minority was still not keen as they perceived themselves to be healthy and at low risk in view of their lifestyle habits, the lack of symptoms and lack of family history of cancer.

“At that point when I was being recruited, I have not decided. Thereon, I read the brochure and I read more. Thereafter, I sort of decided which option I would go for.” P5, tertiary educated homemaker

#### Information sharing

It was recurrently mentioned during the FGDs that participants’ main sources of information came from their friends and family. Many shared of how personal accounts of friends and family had a direct impact on them. In the same way, participants were willing to share the DA with family members, in particular immediate family members such as their spouse and children. Some had reservations sharing with friends, preferring to only share with close friends out of fear of offending others. Some felt uncomfortable talking to friends about topics related to cancer and feces.

“Share with family, my children, my husband, my close friends.” P9, homemaker, when asked how she would make use of the DA

“I think I would not be willing to share with friends… too personal. They might feel offended also. Unless you know them very well, buddies or whatever. I think maybe not… spouse straightaway” P21, tertiary educated

## Discussion

The DA-prototype was generally well received by the participants based on their perspectives on the content, language and visuals. The amount of information was regarded as appropriate and relevant in helping them make a decision. They appreciated being told of all the available screening options, especially the risks involved. Despite this, there were multiple suggestions for improvements. The investigators implemented changes to further contextualize the DA-prototype in accordance with the principles outlined in the Hola Doctor Methodology by Schroeder [30]. (see Additional file 2: Appendix 2: revised DA).

In Singapore, colonoscopies are performed by specialists in tertiary healthcare facilities. In order to qualify for subsidized fees, patients need to be referred by a primary care provider within the public healthcare system. Practical information on the referral process was added to inform users who may not be familiar with the referral process. To better cater to the local setting, terms like “doctor’s office” were replaced with “clinic”. Additional details of IBD and family history of CRC were added as supplementary information.

Participants have expressed difficulty in understanding the bipolar scale. This prompted further literature review which suggests there is evidence, though limited, that bipolar rating scales have lower reliability than unipolar scales [31]. Hence, it was replaced by a unipolar scale, which users can rate from “not important” to “extremely important”. Users who rate more statements as “very important” or “extremely important” will be advised to undertake the FOBT.

The investigators had some concerns that participants may respond adversely to the length and verbosity of the DA prototype. These concerns proved unfounded as most participants found it appropriate and even sought additional information.

As individuals had personal questions, a reminder to clarify their queries with their physicians was highlighted at the end of the DA. Based on the participants’ feedback, editions in languages such as Mandarin, Malay and Tamil will be created at the next stage of development. The favourably perceived layout, font and font size of the DA-prototype were preserved. Even though it is important to keep up with advancements in technology which includes the digitizing of DAs, investigators need to be mindful that in order to reach out to all strata of target users, it is essential that hard copies are made available for those who are less tech savvy.

While participants welcomed the inclusion of visuals, investigators recognised the importance of exercising care and cultural awareness in selecting appropriate visuals. A Muslim participant expressing modesty concerns when viewing a graphic depiction of a colonoscope being inserted illustrated this point. Upholding modesty in dressing is strongly advocated in certain Asian cultures, especially those of Muslim faith who may consider exposure of the body a barrier to seeking healthcare [32]^33^. In multi-ethnic Singapore, it is vital for healthcare providers to be culturally sensitive to avoid compromising the quality of care rendered to specific ethnic groups. The same degree of respect for cultural differences should be extended to the development of the DA. This is similar to the finding in a study by Tan et al. [34] in their cultural adaptation of a DA on insulin therapy, in which Muslim patients’ concern about the non-halal source of insulin was addressed.

In revising the DA, the visuals of the CRC screening options were replaced with hand-drawn graphics. A drape was added to the visual on colonoscopy to minimize the exposure of the gluteal region. These hand-drawn graphics were added to the cover page of the DA for consistency. The visual depicting the steps for using the FIT kit was enlarged for better clarity. Participants were keen to be involved in decision making and identified the doctor as the key healthcare professional to advocate and advise on CRC screening. A trusting doctor-patient relationship is the cornerstone in SDM [35]. It is imperative for doctors to modify their consultation style from a paternalistic approach to SDM. The DA will become a handy tool to facilitate the change. Despite this, studies have demonstrated substantial roles played by other healthcare professionals including nurses, pharmacists and social workers in facilitating SDM [36]. This is particularly relevant where multidisciplinary team-based care is increasingly implemented in healthcare institutions. The use of DA by these paramedical personnel awaits further evaluation.

The willingness of the participants to share the DA with their friends and families suggests the potential utility of the DA beyond the first users. Healthcare professionals should facilitate the spread of the DA to their family members and network of close friends. The investigators intend to place a Quick Response (QR) code on the DA to facilitate information sharing.

Overall, participants self-reported an increase in knowledge and intent for screening after going through the DA. These are important outcomes even for a DA-prototype.

### Strength and limitations

The major ethnic and target age groups in Singapore were represented among the diverse group of participants in this study. This ensured that voices across different segments of society were heard, undoubtedly leading to a more refined DA. This study also reaffirms that cultural adaptation is an efficient process of producing contextualized DAs ready for implementation without compromising on content or acceptability.

While FGDs offer advantages such as richer and wider range of insights being gathered, it is also prone to conformity and emergence of dominant voices. During FGDs, the moderator laid down ground rules that every participant would be provided with ample time and opportunity to express their opinions in order to mitigate these limitations. Additionally, the moderator maintained a neutral stance throughout the FGD, allowing each participant to share their views on the DA content and design without interference, fear and coercion. Those who were quieter were noted by the moderator and invited to speak up on their perspectives, thus ensuring that every participant contributed to the discussion. The moderator continually challenged participants to reflect on views expressed by others from previous FGDs. Any disagreement would be recorded in the interview and presented for discussion during the data analysis and result interpretation.

Another limitation was that member checking was not conducted due to constraint of the grant timeline. The investigators also recognised that the feedback originated from participants with higher educational levels compared to the general population. Further research and modifications of the DA may be necessary to cater to those of lower literacy levels.

### Implications for future research or clinical practice

Of the four phases of cultural adaptation identified by Chenel et al. [22], this study has completed the exploration and adaptation phase. The next phase involves getting feedback from doctors and other healthcare professionals on the DA as they can be advocates for CRC screening. Understanding their perspectives and willingness to use the DA will be a key step towards successful implementation of the tool for SDM. The effectiveness of the revised DA in increasing the CRC screening rate can be subsequently assessed in a RCT. The DA will be translated to other local languages such as mandarin and malay to widen the pool of end users. The DA will be registered with the local Ministry of Culture, Community and Youth in the Singapore Government for official approval to introduce the DA for routine clinical use in the local community. It will be regularly updated to keep abreast with the latest figures and research. The investigators plan to digitalize the DA, so that edits can be rolled out promptly.

## Conclusions

Understanding the users’ perspectives has led to a culturally adapted novel DA with its content and design suited for English-literate Asians. This DA will be introduced in clinical practice to determine its effectiveness in enhancing SDM and increasing CRC screening rate in the local community.

## Supplementary Information

**Additional file 1: Appendix 1**. Topic guide used for in depth interviews and focus group discussions.

**Additional file 2: Appendix 2**. Revised colorectal cancer screening decision aid.

## Data Availability

Qualitative data is not available due to clause in CIRB approval. Refer to appendix for the revised DA. Kindly seek permission from the corresponding author if any authors plan to duplicate the DA and to acknowledge SingHealth Polyclinics (SHP) if they wish to use the DA in their publications.

## Abbreviations

CRC: Colorectal cancer
CT: Computed tomographic
DA: Decision aid
FIT: Faecal immunochemical test
FOBT: Faecal occult blood test
FGD: Focus group discussion
IDI: In depth interview
IBD: Inflammatory bowel disease
IPDAS: International Patient Decision Aid Standards
ODSF: Ottawa Decision Support Framework
SDM: Shared decision making
