# Supplementary material for: Development of a decision aid to support colorectal cancer screening: perspectives of Asians in an endemic urban community—a qualitative research study
Source: BMC Med Inform Decis Mak. 2021 Mar 6;21:86. doi: 10.1186/s12911-021-01404-1 (PMC7936439; doi:10.1186/s12911-021-01404-1)
Supplement: Supplementary file 1 — Additional file 1: Appendix 1. Topic guide used for in depth interviews and focus group discussions. [file 12911_2021_1404_MOESM1_ESM.pdf]

Supplementary Appendix 1

Topic Guide
- How do you find the layout of the patient decision aid?<br>- How do you find the font and font sizes?<br>- How do you find the diagrams?
- What do you think is the purpose of this colorectal cancer screening patient decision aid?<br>- How do you feel about being given a choice about the various options of colorectal cancer screening?<br>- How do you feel about being told the risks and benefits of colorectal cancer screening options?<br>- Is the information provided easy to understand?<br>- Does the patient decision aid provide too much or too little information for you to make a decision? Please elaborate.
- Does the patient decision aid address the concerns you have regarding colorectal cancer screening? Please elaborate.<br>- How has the patient decision aid affected your decision for colorectal cancer screening?<br>- How will you make use of the patient decision aid?
