# Supplementary material for: Development of a decision aid to support colorectal cancer screening: perspectives of Asians in an endemic urban community—a qualitative research study
Source: BMC Med Inform Decis Mak. 2021 Mar 6;21:86. doi: 10.1186/s12911-021-01404-1 (PMC7936439; doi:10.1186/s12911-021-01404-1)
Supplement: Supplementary file 2 — Additional file 2: Appendix 2. Revised colorectal cancer screening decision aid. [file 12911_2021_1404_MOESM2_ESM.pdf]

Thank you for using this booklet.
We hope you find it useful.

This decision aid is not meant to replace the consultation.

Whatever you decide, please clarify your queries and discuss your opinion(s) with your health care provider.

Authors:

Dr Yuen Sok Wei Julia, Dr Tay Tsang Yew,
Ms Gao Ning, Dr Tho Nian Qin,
Clinical Assoc Prof (Dr) Tan Ngiap Chuan

Principal Investigator:

Dr Yuen Sok Wei Julia

All authors have declared no conflict of interest.

Reference:

Singapore Cancer Society 2016
(https://www.singaporecancersociety.org.sg/learn-about-cancer/types-of-cancer/colorectal-cancer.html)

Polyclinics
SingHealth

PATIENTS. AT THE HEART OF ALL WE DO.®

ISDN 000000

Current as of: April 2020.

WHICH COLORECTAL CANCER SCREENING TEST SHOULD I CHOOSE?

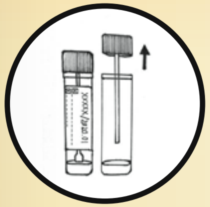

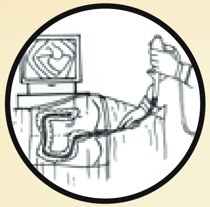

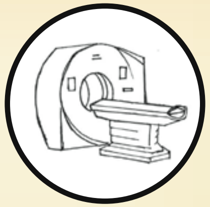

Polyclinics
SingHealth

Hello!

Your healthcare provider has just told you about colorectal cancer screening.

This booklet will help to address some of your concerns and assist you with making a decision regarding colorectal cancer screening.

1. Introduction to colorectal cancer screening

Why is colorectal cancer screening important?

- Colorectal Cancer is the most common cancer diagnosed in Singapore.
- Regular screening can pick up colon polyps which can be removed to prevent development of cancer.
- It can also detect early colorectal cancer which is easier to treat.

9,807

new cases of colorectal cancer diagnosed from 2011-2015

About **3 men** and **2 women** are diagnosed with colorectal cancer **daily!**

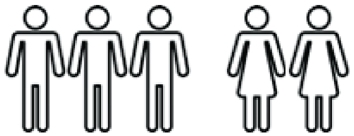

What is colorectal cancer?

- Colorectal cancer is cancer of the colon and rectum.
- It usually begins with polyps which are growths on the inner lining of the colon or rectum.
- Most polyps do not turn cancerous.
- However, certain types of polyps can develop into cancer over time and should be removed if detected.

What is colorectal cancer screening?

- Colorectal cancer screening is recommended even if you feel well and have no symptoms.
- This is because early stages of colorectal cancer often have no signs or symptoms.
- You should see your doctor early if you have the following symptoms which need further evaluation.

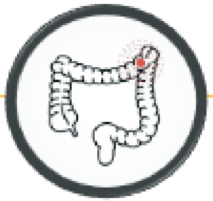

See a doctor early if you have the following signs and symptoms of colorectal cancer.

Change in bowel habits, unexplained weight loss, abdominal pain or discomfort and bleeding from the anus.

Who should be screened?

- Everyone aged 50 years and above should be screened.
- However, if you have the following risk factors, you are at a higher risk and should see your healthcare provider to discuss your options.
  - Family history of colorectal cancer (especially first degree relatives such as parents or siblings)
  - Personal history of colorectal cancer
  - History of inflammatory bowel disease (Example: Crohn's disease and Ulcerative colitis)

2. What are your options?

Individuals at average risk have a variety of screening options to choose from:

No screening

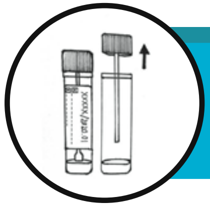

Stool Test,
also known as the Faecal Occult Blood Test (FOBT)

Colonoscopy

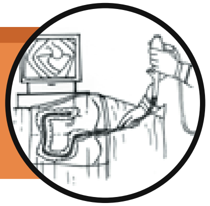

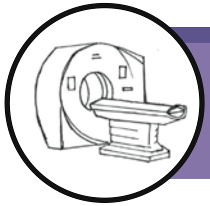

Others:
- Computed Tomographic (CT) scan of the colon
- Flexible Sigmoidoscopy

Option 1

Stool Test, also known as Faecal Occult Blood Test (FOBT)

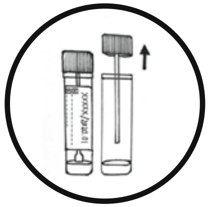

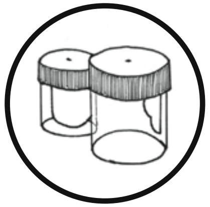

- Polyps and cancers in the colon and rectum can bleed
- FOBT tests for small amounts of blood in the stool that cannot be seen
- Can be done at home at your own time
- Perform the tests twice (2 specimens on 2 separate days)
- Should be done annually
- Can be done via kits obtained from Singapore Cancer Society or kits available commercially

1. User guide for the stool test from Singapore Cancer Society

- The FIT KIT is free
- Singaporeans or Permanent Residents aged 50 years old and above are eligible
- FIT Kit collection points:
  - Singapore Cancer Society Main Office
  - Guardian Health and Beauty stores
  - Watson Personal Care stores
  - Eu Yan Sang

Each participant will receive 2 FIT Kits and is required to perform 1 test each on 2 consecutive days or any 2 days within 1 week.

How to use the FIT kit

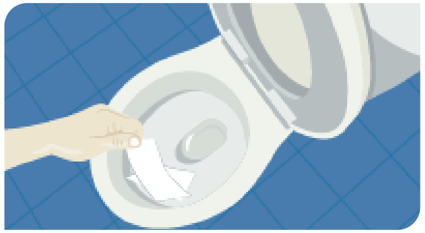

1. Collect your stool on clean toilet paper. The stool must not come into contact with water or urine.

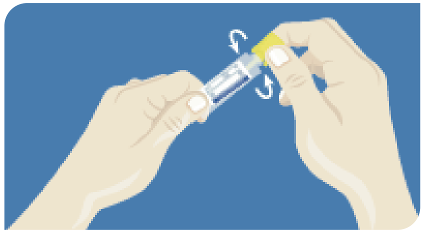

2. Twist and pull the tab to take out the sampling probe from the FIT Kit.

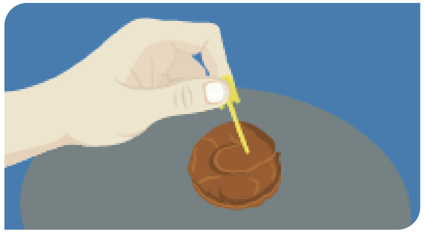

3. Poke the probe into 6 different areas of the stools.

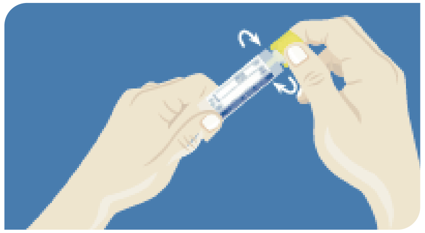

4. Return the sampling probe back to the FIT Kit and secure the tab by twisting it to close it. Shake the kit several times.

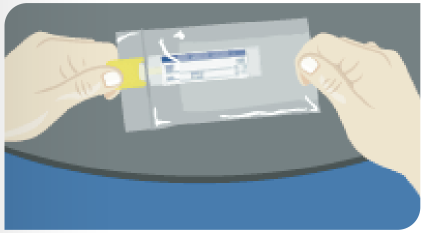

5. Use the absorbent pad to wrap around the FIT Kit and place them into the ziplock bag provided. Squeeze the excess air out and seal securely.

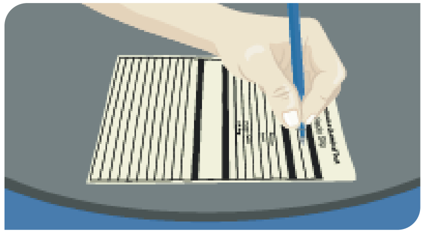

6. Ensure that all your particulars are correct and the stool collection date is clearly written on the reply slip provided.

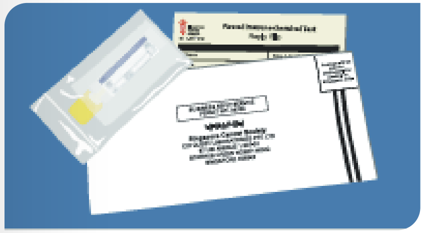

7. Mail the FIT Kit together with the reply slip back to Singapore Cancer Society, using the business reply envelope provided within 2 days of collecting your stool specimen.

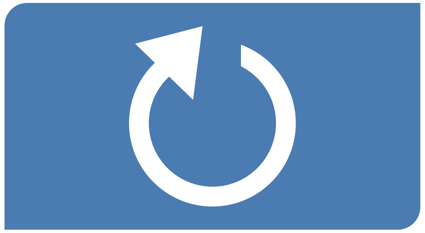

8. Repeat steps 1 to 7 and collect the second sample with the second FIT Kit.

Diagram courtesy of Singapore Cancer Society
https://www.singaporecancersociety.org.sg/get-screened/colorectal-cancer/fit-kit.html#user-guide

2. User guide for the stool test from polyclinic

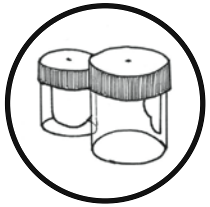

1. You will be given 2 stool specimen bottles

2. Collect your stool on clean toilet paper (can be done at home at your own time)

3. The stool must not come into contact with water or urine

4. Twist and remove the lid from the bottle

5. Collect some stool sample using the spoon attached to the lid

6. Repeat the above steps on another day to collect the 2nd stool sample

7. Return both stool specimen bottles to the polyclinic lab

Option 2

Colonoscopy

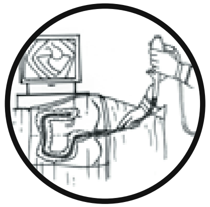

- The most accurate assessment of the colon and the gold standard for diagnosis
- Direct visualisation and examination of the colon and rectum by an endoscope inserted via the anus
- Allows for removal of polyps
- Has to be done by a specialist in a clinic/ hospital setting
- Performed once every 10 years
- Requires you to take a low residue diet three days before the colonoscopy
- Requires you to take clear soup/ drinks for dinner the night before colonoscopy
- Requires bowel preparation the night before colonoscopy: drinking laxative solution and having to go to the toilet multiple times to clear out the bowels
- Requires sedation to perform the test

Option 3

Others

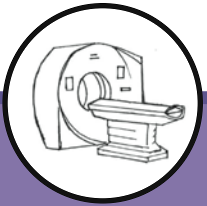

Computed Tomographic (CT) scan of the colon

- Minimally invasive imaging examination of the colon and rectum using CT scan
- Performed once every five years

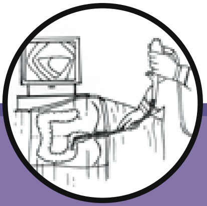

Flexible Sigmoidoscopy

- Similar to colonoscopy but only examines lower half of the colon
- Requires an enema before the procedure
- Performed once every five years

3. Comparing your options

What are the pros and cons?

	What is usually involved?
**No screening**	• Monitor for signs and symptoms of colorectal cancer<br>• See a doctor early if you experience any symptoms
**Stool test/ FOBT**	• Done once every year<br>• Collect stool samples at home, 2 samples on 2 different days<br>• Mail the sample to the lab or return to your doctor's clinic<br>• If test is positive, you will need a colonoscopy
**Colonoscopy**	• Done once every 10 years (may be done more frequently depending on findings)<br>• Test done in a specialist's clinic or hospital as a day procedure
**Others: CT scan of colon/ flexible sigmoidoscopy**	• Done once every 5 years<br>• Test done in a specialist's clinic or hospital as a day procedure

	What is the cost?	What are the benefits?	What are the risks and side effects?
**No screening**	There is no additional cost	• You need not have to go through the stress and anxiety of having a positive stool test<br>• Less than 10 out of 100 people without colorectal cancer will have a positive FOBT	• Delay early detection and treatment of pre-cancerous polyps or colorectal cancer
**Stool test/FOBT**	• FIT kits provided by Singapore Cancer Society are free of charge<br>• Stools tests done at polyclinics are about $6-7*<br>• Cheapest compared to other options	• Screening lowers your risk of getting and dying from colorectal cancer<br>• 4-6 out of 100 people who tested positive had colorectal cancer<br>• It's easy to do at home at your convenience<br>• You do not need to use laxatives	• If the test is positive, a colonoscopy will be required to rule out presence of pre-cancerous polyps or colorectal cancer
**Colonoscopy**	• Cash payment of about $50-$650* after government subsidy and medisave claim for Singaporeans and PRs	• Screening lowers your risk of getting and dying from colorectal cancer<br>• Precancerous polyps can be removed during the procedure<br>• You do not have to do the test as often as the stool test	• Requires bowel preparation<br>• Risk of sedation<br>• Risk of procedure - tears in the colon (1 in 2500 procedures) and major bleeding (1 in 1250 procedures) requiring emergency operation
**Others: CT scan of colon/ flexible sigmoidoscopy**	**CT scan of colon**<br>• Cash payment of about $300-$600* after government subsidy and medisave claim for Singaporeans and PRs<br>**Flexible sigmoidoscopy**<br>• Cash payment of about $100-$400* after government subsidy and medisave claim for Singaporeans and PRs	• Screening lowers your risk of getting and dying from colorectal cancer<br>• You do not have to do the test as often as the stool test	**CT scan of colon**<br>• Requires bowel preparation<br>• Requires ingestion of contrast medium<br>• May miss polyps less than 1cm<br>• Exposes you to some radiation<br>**Sigmoidoscopy**<br>• Requires an enema<br>• Only examines the left side of the colon, may miss lesions on the right side of the colon

*Estimated costs at time of print.

4. What matters most to you?

Your feelings are just as important as medical facts

✓ Tick the appropriate box

How important is this to you?	Not important	Slightly important	Important	Very important	Extremely important
Being able to do the test at home in private rather than going to a clinic/ hospital					
Being able to do the test at a time of your convenience rather than taking time off to go to a clinic/ hospital					
Doing the test once every year instead of once every 5 to 10 years					
Not having to drink laxative solution and spend a lot time in the toilet to clear your bowels					
The potential risks of undergoing colonoscopy/ other procedure					
The higher cost of colonoscopy/ other procedure					

If you have ticked more boxes under Very/ Extremely Important, consider doing the stool test (FOBT)

If you have ticked more boxes under Not/ Slightly Important, consider doing the colonoscopy/ other procedure

5. What is your decision?

Are you ready to make a decision for screening?

Yes, I want to do the Stool Test

- ☐ Collect your free FIT kit from collections points (refer to page 5)

  OR

- ☐ Visit your primary healthcare provider

Yes, I want to do the Colonoscopy or others

- ☐ Visit your primary healthcare provider to obtain a referral letter for your test in hospital

Undecided

- ☐ It's ok if you are not ready to make a decision

  We encourage you to continue to discuss with your healthcare providers.
